# The Mental Health Status and Associated Factors Among Medical Students Engaged in Online Learning at Home During the Pandemic: A Cross-Sectional Study From China

**DOI:** 10.3389/fpsyt.2021.755503

**Published:** 2021-12-23

**Authors:** Wei-wei Chang, Liu-xia Shi, Liu Zhang, Yue-long Jin, Jie-gen Yu

**Affiliations:** ^1^Department of Epidemiology and Health Statistics, School of Public Health, Wannan Medical College, Wuhu, China; ^2^Department of Oral Medicine, School of Stomatology, Wannan Medical College, Wuhu, China; ^3^Department of Hospital Infection Management Office, Wuhu Hospital of Traditional Chinese Medicine, Wuhu, China; ^4^School of Humanities and Management, Wannan Medical College, Wuhu, China

**Keywords:** COVID-19, depression, anxiety, stress, online learning, medical students

## Abstract

**Background:** The purpose of this study was to assess the mental health status of medical students engaged in online learning at home during the pandemic, and explore the potential risk factors of mental health.

**Methods:** A cross-sectional study was conducted via an online survey among 5,100 medical students from Wannan Medical College in China. The Depression, Anxiety and Stress scale (DASS-21) was used to measure self-reported symptoms of depression, anxiety, and stress among medical students during online learning in the pandemic.

**Results:** In total, 4,115 participants were included in the study. The prevalence symptoms of depression, anxiety, and stress were 31.9, 32.9, and 14.6%, respectively. Depression was associated with gender, grade, length of schooling, relationship with father, students' daily online learning time, and students' satisfaction with online learning effects. Anxiety was associated with gender, length of schooling, relationship with father, relationship between parents, students' daily online learning time, and students' satisfaction with online learning effects. Stress was associated with grade, relationship with father, relationship between parents, students' daily online learning time, and students' satisfaction with online learning effects.

**Conclusions:** Nearly one-third of medical students survived with varying degrees of depression, anxiety, and stress symptoms during online learning of the COVID-19 pandemic. Gender, grade, length of schooling, family environment, and online learning environment play vital roles in medical students' mental health. Families and schools should provide targeted psychological counseling to high-risk students (male, second-year and third-year, four-year program). The findings of this study can provide reference for educators to cope with the psychological problems and formulate the mental health curriculum construction among medical students during online learning.

## Introduction

Since December 2019, the coronavirus disease 2019 (COVID-19) has spread rapidly in China and around the world (1). As of April 5, 2020, there were more than 1.20 million diagnosed cases and more than 68,000 deaths in 215 countries and regions around the world (2). The WHO classified COVID-19 as a pandemic on March 11, 2020 (3). As of 24:00, May 31, 2020, a total of 83,001 COVID-19 cases had been confirmed and 4,634 people had died due to COVID-19-related illnesses in China (4).

COVID-19 is a respiratory infectious disease with strong transmission intensity (5). In order to control the spread of the epidemic, strict traffic control has been implemented throughout China, and a large number of university students have been implemented strict self-isolation in their hometowns (6). In order to solve the problem of students unable to study in school, the Department of Education of Anhui Province (China) issued a notice On February 24, 2020: all kinds of schools (universities, middle schools, etc.) in Anhui Province postponed the opening of classes, and implemented the online education on March 2, 2020 (7). In response to the Ministry of Education' s call for “Disrupted classes, undisrupted learning,” teachers have begun to prepare for online learning from mid-February. For the first time, the teaching and learning of all courses has completely changed from offline to online, which is undoubtedly a severe test for college students who are used to classroom learning (8).

As a global public health event, COVID-19 can lead to psychological crises such as acute stress disorder, post-traumatic stress disorder (PTSD), anxiety, and depression (9). Several studies have indicated that the outbreak of infectious diseases causes mental health problems (10–13). After the occurrence of severe acute respiratory syndrome (SARS), the positive detection rate of PTSD in first-line medical workers was 25.8%, while the total score of anxiety and depression of medical students was significantly higher than that of medical workers (14). During the COVID-19 pandemic, the mental health of patients and front-line medical staff has received significant attention (10, 15). Medical students have more academic pressure, and they are at higher risk of mental diseases, such as anxiety and suicidal ideation (16, 17). As a new force in the medical field, medical students have insufficient clinical experience and no opportunity to participate in the front-line anti-epidemic work. Ye et al. reported that medical students were suffering from more stress than non-medical students (18). During the period of home isolation, medical students have reduced social contact and support from peers (6, 19). A systematic review from 100 eligible studies indicated that social support was associated with depression protection, and parental support was the most important among adolescents (20). During the period of home isolation, the COVID-19 pandemic had a negative impact on the mental health of college students (21, 22). For college students who are used to classroom learning, online teaching may have a certain impact on their psychology (23). The study studied by Zhang et al. indicated that the percentage of learning burnout among medical students engaged in online learning was as high as 46.05% (24). The above researches suggests that medical students engaged in online learning during the epidemic might be more prone to develop negative psychological, owning to face the double pressure of the pandemic and online learning.

Therefore, mental health of medical students engaged in online learning should cause concern. This study first investigated the mental health of medical students during online learning after the outbreak of COVID-19 in China, and further examined the potential risk factors of mental health. The results of this study can provide a theoretical basis for psychological intervention for medical students. This study will also strengthen the impact of medical education and provide reference for better creating innovative online teaching strategies among medical students.

## Methods

### Participants and Procedure

The participants of this study were medical students who participated in online learning in the 2019/2020-2 semester from Wannan Medical College in Anhui Province, China. The school has a total of 15,813 students in the 2019/2020-2 semester, including 18 majors with four-year programs and 7 majors with five-year programs. Different majors include 2–30 classes, with about 25–33 students in each class. Students in grades 4 and 5 participated in their internship and did not need to partake the school's online learning. All students in grades 1–3 (10,923 students) participated in online learning. Using stratified random cluster sampling method, 5,100 students in 170 classes were selected as the participants. The specific implementation method was as follows: (1) Firstly, we stratified the students according to the length of schooling (four-year programs and five-year programs), and then stratified according to the majors; (2) Secondly, 40–50% of classes in each grade (grade 1–3) were randomly selected from different majors; and (3) Finally, the researcher contacted the counselors of each grade in different majors to count the numbers of students in the selected class.

As the students were required to quarantine online learning at home, a web-based survey was distributed through the Wenjuan platform (https://www.wenjuan.com/list/). Before the formal survey, we selected 10 students to conduct an online pre-survey. According to the results of students' feedback, the questionnaire was revised and improved. The formal survey period was from June 1 to June 15, 2020. The counselor sent the online questionnaire to the students via WeChat and QQ. The purpose of the online survey and the precautions for filling in the online questionnaire was told to the students by the counselor. Students were informed that they could participate in the survey voluntarily. The survey took about five min on average to complete. This study was approved by School of Public Health of Wannan Medical College (LL-2020BH2086).

A total of 4,356 online questionnaires surveys were collected. However, 241 participants were excluded from analysis because they took too little time (5–30 s) to complete the questionnaire. Finally, 4,115 respondents were included in the final analysis (80.69% response rate: 4115/5100).

### Survey Instrument

The structured questionnaire included the following four aspects: basic information, online learning, family factors, and the mental health of students.

The basic information included gender (male, female), age, grade (first-year, second-year, third-year), length of schooling (four-year program, five-year program), birthplace (city, town, village), father's education level (primary school or below, junior high school, senior high school, junior college, bachelor degree and above), mother's education level (primary school or below, junior high school, senior high school, junior college, bachelor degree and above), the records of students in the class in the past six months (top-grade, middle-grade, bottom-grade).

The online learning-related information included students' daily online learning time (0–2 h, 2–4 h, ≥4 h) and students' satisfaction with online learning effects (satisfied, general, dissatisfied). Family factors included the relationship between parents (good, general, poor), relationship with mother (good, general, poor), and relationship with father (good, general, poor).

The Chinese version of the 21-item Depression Anxiety Stress Scale (DASS-21) was used to measure the mental health of medical students (25). Each item was scored on a 4-point Likert scale from 0 (did not apply to me at all) to 3 (applied to me very much or most of the time). A total of 21-items included three subscales: depression (questions 3, 5, 10, 13, 16, 17, and 21), anxiety (questions 2, 4, 7, 9, 15, 19, and 20), and stress (questions 1, 6, 8, 11, 12, 14, and 18). The three subscales were divided into five levels as follows: depression (normal, 0–9 scores; mild, 10–13 scores; moderate, 14–20 scores; severe, 21–27 scores; extremely severe, 28–42 scores), anxiety (normal, 0–7 scores; mild, 8–9 scores; moderate, 10–14 scores; severe, 15–19 scores; extremely severe, 20–42 scores), and stress (normal, 0–14 scores; mild, 15–18 scores; moderate, 19–25 scores; severe, 26–33 scores; extremely severe, 34–42 scores) (26). The scores of DASS 21 were divided into two groups of normal and symptomatic (mild/moderate/severe/extremely severe). The DASS-21 has good reliability and validity, which has been widely used in college students (27–29). In present study, the Cronbach's α for the depression, anxiety, and stress subscale was 0.896, 0.859, and 0.873, respectively, indicating a good internal consistency for each subscale.

### Data Analysis

Data were analyzed using SPSS 26.0 software. Descriptive statistics were performed to reflect the basic characteristics of students using mean (M) and standard deviation (SD) for quantitative data and percentages for qualitative data.

Depression, anxiety, and stress subscale scores were classified as normal and symptomatic (26). A Chi-square test which was used to screen the candidate variables affecting the mental health of medical students was performed to compare the prevalence of symptoms of depression, anxiety, and stress in two (gender, length of schooling) or more groups (grade, birthplace, father's education level, mother's education level, the records of students in the class in the past six months, students' daily online learning time, students' satisfaction with online learning effects, the relationship between parents, relationship with mother, and relationship with father).

The variables with *P* ≤ 0.1 in the univariate analysis (a Chi-square test) were included in the multivariate regression model: 9 variables for depression (gender, grade, length of schooling, the relationship between parents, relationship with mother, relationship with father, the records of students in the class in the past six months, students' daily online learning time, and students' satisfaction with online learning effects), 8 variables for anxiety (gender, length of schooling, the relationship between parents, relationship with mother, relationship with father, the records of students in the class in the past six months, students' daily online learning time, and students' satisfaction with online learning effects), and 10 variables for stress (gender, grade, length of schooling, father's education level, the relationship between parents, relationship with mother, relationship with father, the records of students in the class in the past six months, students' daily online learning time, and students' satisfaction with online learning effects).

Finally, taking depression, anxiety, and stress (binary dependent variables: normal vs. symptomatic) as dependent variables, the multivariate logistic regression analysis (forward method, 95% confidence interval) was used to explore the potential associated factors of mental health among medical students. A value of *P* < 0.05 (two-tailed) was considered statistically significant.

## Results

### Basic Characteristics of Medical Students

Among 4,115 medical students, 1,626 (39.51%) were males, and ages ranged between 17 and 26 years (20.27 ± 1.30 years). 1,328 (32.27%), 1,639 (39.83%), and 1,148 (27.90%) students were from the first, second, and third-year, respectively. In term of length of schooling, five-year and four-year accounted for 56.82% and 43.18%, respectively. More than half (*n* = 2,105, 51.15%) students were located in village.

Most of students reported that, they had a good relationship with mother (86.56%) and father (74.73%), and their parents had a good relationship (77.47%). With respect to students' daily online learning time, 2,971 (72.20%) students studied for 4 h or more, 794 (19.30%) studied for 2–4 h, and 350 (8.50%) students studied for <2 h. Lower than half of students (36.45%) satisfied with the effect of online teaching, and 48.19% of students were generally satisfied with online teaching. Other basic characteristics was shown in Table 1.

**Table 1:** The characteristics of research participants (*n* = 4,115).

**Variables**	**Group**	**Number**	**Constituent ratio (%)**
Gender	Male	1,626	39.51
	Female	2,489	60.49
Age (year), mean ± SD	20.27 ± 1.30, Range (17–26)		
Birthplace	City	943	22.92
	Town	1,067	25.93
	Village	2,105	51.15
Grade	First-year	1328	32.27
	Second-year	1,639	39.83
	Third-year	1,148	27.90
Father's education level	Primary school or below	763	18.54
	Junior high school	2,057	49.99
	Senior high school	738	17.93
	Junior college	311	7.56
	Bachelor degree and above	246	5.98
Mother's education level	Primary school or below	1,689	41.04
	Junior high school	1,586	38.54
	Senior high school	508	12.35
	Junior college	225	5.47
	Bachelor degree and above	107	2.60
Relationship between parents	Poor	155	3.77
	General	772	18.76
	Good	3,188	77.47
Relationship with mother	Poor	36	0.88
	General	517	12.56
	Good	3,562	86.56
Relationship with father	Poor	107	2.60
	General	933	22.67
	Good	3,075	74.73
The school records in the class in the past six months	Top-grade	1,500	36.45
	Middle-grade	1,851	44.98
	Bottom-grade	764	18.57
Online learning time (hours)	0–2	350	8.50
	2–4	794	19.30
	≥4	2,971	72.20
Satisfaction with online learning effects	satisfied	1,514	36.79
	General	1,983	48.19
	Dissatisfied	618	15.02

### Mental Health of Medical Students Engaged in Online Learning at Home During the Pandemic

The overall mean score for each of the DASS-21 subscales was 6.66 ± 7.17 for depression symptoms, 5.73 ± 6.34 for anxiety symptoms, and 8.36 ± 7.20 for stress symptoms. 31.9, 32.9, and 14.6% of participants were at symptomatic levels (mild to extremely severe) according to depression (scores ≥10), anxiety (scores ≥8), and stress scores (scores ≥15), respectively.

The prevalence of depression symptoms at mild, moderate, severe, and extremely severe levels were 11.1, 16.4, 2.6, and 1.8%, respectively, and depression symptoms were mainly at moderate level (16.4%). The prevalence of anxiety symptoms at mild, moderate, severe, and extremely severe levels were 6.6, 19.0, 3.5, and 3.7%, respectively, and anxiety symptoms were mainly at moderate level (19.0%). 7.7, 4.3, 1.9, and 0.8% of participants were at mild, moderate, severe, and extremely severe levels, respectively, and stress symptoms were mainly at mild level (7.7%) (Table 2).

**Table 2:** Distribution of all subjects' grades in each DASS-21 subscale [*n* (%)] (*n* = 4,115).

**Group**	**Depression**	**Anxiety**	**Stress**
No symptoms	2,803 (68.1)	2,763 (67.1)	3,515 (85.4)
Mild	457 (11.1)	273 (6.6)	317 (7.7)
Moderate	674 (16.4)	782 (19.0)	175 (4.3)
Severe	107 (2.6)	146 (3.5)	77 (1.9)
Extremely severe	74 (1.8)	151 (3.7)	31 (0.8)

### Factors Influencing Medical Students' Mental Health During the Pandemic Online Learning at Home

#### Univariate Analysis

The prevalence of symptoms of depression, anxiety, and stress among males were higher than females (36.59 vs. 28.80%; 38.01 vs. 29.50%; 15.74 vs. 13.82%, respectively) (χ^2^ = 27.454, 32.344, and 2.921; *P* < 0.001, *P* < 0.001, and *P* = 0.087, respectively). The prevalence of symptoms of depression and anxiety among students whose length of schooling is four-year were higher than those of five-year (34.33 vs. 30.03%; 35.23 vs. 31.05%, respectively) (χ^2^ = 8.603 and 7.980; *P* = 0.003 and *P* = 0.005, respectively) (Table 3).

**Table 3:** Comparison of mental health levels among medical students with different characteristics [*n* (%)].

**Variables**	**Group**	**Depression**	**Anxiety**	**Stress**
Gender	Male (*n* = 1,626)	595 (36.59)	618 (38.01)	256 (15.74)
	Female (*n* = 2,489)	717 (28.80)	734 (29.50)	344 (13.82)
	χ^2^	27.454	32.344	2.921
	*P*	<0.001	<0.001	0.087
Length of schooling	Five-year	702 (30.03)	726 (31.05)	339 (14.50)
	Four-year	610 (34.33)	626 (35.23)	261 (14.69)
	χ^2^	8.603	7.980	0.029
	*P*	0.003	0.005	0.866
Grade	First-year (*n* = 1,328)	383 (28.84)	415 (31.25)	140 (10.54)
	Second-year (*n* = 1,639)	554 33.80)	561 (34.22)	267 (16.29)
	Third-year (*n* = 1,148)	375 (32.66)	376 (32.75)	193 (16.81)
	χ^2^	8.761	2.957	25.825
	*P*	0.013	0.228	<0.001
Birthplace	City (*n* = 943)	299 (31.70)	295 (31.28)	140 (14.85)
	Town (*n* = 1,067)	325 (30.46)	357 (33.46)	150 (14.05)
	Village (*n* = 2,105)	688 (32.68)	700 (33.25)	310 (14.72)
	χ^2^	1.631	1.384	0.323
	*P*	0.442	0.501	0.851
Father's education level	Primary school or below (*n* = 763)	258 (33.81)	264 (34.60)	128 (16.78)
	Junior high school (*n* = 2,057)	644 (31.31)	661 (32.13)	274 (13.32)
	Senior high school (*n* = 738)	230 (31.17)	249 (33.74)	108 (14.63)
	Junior college (*n* = 312)	106 (33.97)	99 (31.73)	43 (13.78)
	Bachelor degree and above (*n* = 245)	74 (30.20)	79 (32.25)	47 (19.18)
	χ^2^	2.745	2.02	9.905
	*P*	0.601	0.732	0.042
Mother's education level	Primary school or below (*n* = 763)	563 (33.33)	584 (34.58)	254 (15.04)
	Junior high school (*n* = 2,057)	482 (30.39)	494 (31.14)	224 (14.12)
	Senior high school (*n* = 738)	158 (31.10)	163 (32.09)	70 (13.78)
	Junior college (*n* = 312)	80 (35.56)	75 (33.33)	34 (15.11)
	Bachelor degree and above (*n* = 245)	29 (27.10)	36 (33.64)	18 (16.82)
	χ^2^	5.927	4.555	1.295
	*P*	0.205	0.336	0.862
Relationship between parents	Good (*n* = 3,188)	877 (27.82)	937 (29.39)	386 (12.11)
	General (*n* = 772)	343 (44.43)	336 (43.52)	150 (19.43)
	Poor (*n* = 155)	82 (52.90)	79 (50.97)	64 (41.29)
	χ^2^	116.691	80.214	119.011
	$\chi_{\text{trend}}^{2}$	109.115	78.498	104.781
	*P*	<0.001	<0.001	<0.001
Relationship with mother	Good (*n* = 3,562)	1,034 (29.03)	1,098 (30.82)	483 (13.56)
	General (*n* = 517)	257 (49.71)	232 (44.87)	102 (19.73)
	Poor (*n* = 36)	21 (58.33)	22 (61.11)	15 (41.67)
	χ^2^	100.612	53.536	35.19
	$\chi_{\text{trend}}^{2}$	97.718	53.462	29.606
	*P*	<0.001	<0.001	<0.001
Relationship with father	Good (*n* = 3,075)	834 (27.12)	897 (29.17)	371 (12.07)
	General (*n* = 933)	406 (43.52)	382 (40.94)	184 (19.72)
	Poor (*n* = 107)	72 (67.29)	73 (68.22)	45 (42.06)
	χ^2^	151.991	107.264	100.274
	$\chi_{\text{trend}}^{2}$	150.157	99.429	87.859
	*P*	<0.001	<0.001	<0.001
The school records in the class in the past six months	Top-grade (*n* = 1,500)	385 (25.67)	433 (28.87)	208 (13.87)
	Middle-grade (*n* = 1,851)	582 (31.44)	597 (32.25)	228 (12.32)
	Bottom-grade (*n* = 764)	345 (45.16)	322 (42.15)	164 (21.47)
	χ^2^	88.84	41.019	37.305
	$\chi_{\text{trend}}^{2}$	81.804	36.369	15.091
	*P*	<0.001	<0.001	<0.001
Online learning time (hours)	0–2 (*n* = 350)	165 (47.14)	162 (46.29)	89 (25.43)
	2–4 (*n* = 794)	304 (38.29)	318 (40.05)	142 (17.88)
	≥4 (*n* = 2,971)	843 (28.37)	872 (29.35)	369 (12.42)
	χ^2^	69.364	63.794	51.162
	$\chi_{\text{trend}}^{2}$	69.284	62.681	50.727
	*P*	<0.001	<0.001	<0.001
Satisfaction with online learning effects	Satisfied (*n* = 1,514)	355 (23.45)	395 (26.09)	147 (9.71)
	General (*n* = 1,983)	680 (34.29)	688 (34.69)	279 (14.07)
	Dissatisfied (*n* = 618)	277 (44.82)	269 (43.53)	174 (28.16)
	χ^2^	102.538	66.361	120.696
	$\chi_{\text{trend}}^{2}$	102.503	66.339	102.992
	*P*	<0.001	<0.001	<0.001

The worse the relationship between parents, the relationship with mother, and the relationship with father, the higher the prevalence of symptoms of depression, anxiety, and stress (depression: $\chi_{\text{trend}}^{2}$ = 109.115, 97.718, and 150.157; anxiety: $\chi_{\text{trend}}^{2}$ = 78.498, 53.462, and 99.429; stress: $\chi_{\text{trend}}^{2}$ = 104.781, 29.606, and 87.859; *P* < 0.001). The prevalence of symptoms of depression, anxiety, and stress was higher in the students with the bottom-grade school records in the past half year, compared with the students with the middle- and the top-grades (*P* < 0.001) (Table 3).

As students' satisfaction with online teaching decreased, the prevalence of symptoms of depression, anxiety and stress rose ($\chi_{\text{trend}}^{2}$ = 102.503, 66.339, and 102.992; all *P* < 0.001). The longer the students' online learning time, the lower the prevalence of symptoms of depression, anxiety, and stress ($\chi_{\text{trend}}^{2}$ = 69.284, 62.681, and 50.727; all *P* < 0.001) (Table 3).

#### Multivariate Analysis

Variables with *P* < 0.10 in univariate analysis were included in the multivariate analysis: 9 variables for depression, 8 variables for anxiety, and 10 variables for stress. Taking depression, anxiety, and stress of medical students as dependent variables (0 = No, 1 = Yes), logistic regression analysis was conducted to explore factors associated factors. The risk of depression was significantly increased among medical students with the following variables: males (OR = 1.304, 95% CI: 1.130–1.505), four-year programs (OR = 1.277, 95% CI: 1.105–1.476), grade (second grade: OR = 1.210, 95% CI: 1.021–1.434; third grade: OR = 1.304, 95% CI: 1.081–1.572), relationship with father (poor: OR = 4.706, 95% CI: 3.036–7.296; general: OR = 1.618, 95% CI: 1.339–1.955), the school records in the past six months (bottom: OR = 2.169, 95% CI: 1.784–2.638; middle: OR = 1.301, 95% CI: 1.109–1.526), satisfaction with online learning effects (dissatisfied: OR = 2.308, 95% CI: 1.873–2.844; general: OR = 1.596, 95% CI: 1.365–1.868), and online learning time (0–2 h: OR = 1.779, 95% CI: 1.400–2.259; 2–4 h: OR = 1.398, 95% CI: 1.177–1.660) (Table 4).

**Table 4:** Wald (forward) logistic regression analyses for factors associated with depression, anxiety, and stress of medical students (*n* = 4115).

**Dependent variable**	**Factors**	***B***	***Wald***	***P***	***OR***	**95% CI**	
**Depression**	Gender (Male vs. female)	0.265	13.161	<0.001	1.304	1.130	1.505
	Length of schooling (Four-year vs. Five year) Grade (vs. First-year	0.245	10.998	0.001	1.277	1.105	1.476
	Second-year	0.191	4.827	0.028	1.210	1.021	1.434
	Third-year	0.265	7.704	0.006	1.304	1.081	1.572
	**Relationship with father (vs. Good)**						
	General	0.481	24.802	<0.001	1.618	1.339	1.955
	Poor	1.549	47.938	<0.001	4.706	3.036	7.296
	**The school records in the class in the past six months (vs. Top-grade)**						
	Middle-grade	0.263	10.457	0.001	1.301	1.109	1.526
	Bottom-grade	0.774	60.171	<0.001	2.169	1.784	2.638
	**Satisfaction with online learning effects (vs. Satisfied)**						
	General	0.468	34.131	<0.001	1.596	1.365	1.868
	Dissatisfied	0.837	61.618	<0.001	2.308	1.873	2.844
	**Online learning time (vs**. **≥4 h)**						
	0–2 h	0.576	22.242	<0.001	1.779	1.400	2.259
	2–4 h	0.335	14.579	<0.001	1.398	1.177	1.660
**Anxiety**							
	Gender (Male vs. female)	0.351	24.207	<0.001	1.421	1.235	1.634
	Length of schooling (Four-year vs. Five year)	0.202	8.454	0.004	1.224	1.068	1.403
	**Relationship between parents (vs. Good)**						
	General	0.333	9.147	0.002	1.395	1.124	1.732
	Poor	0.360	3.451	0.063	1.433	0.980	2.094
	**Relationship with father (vs. Good)**						
	General	0.254	6.109	0.013	1.289	1.054	1.576
	Poor	1.283	28.443	<0.001	3.609	2.252	5.784
	**The school records in the class in the past six months (vs. Top-grade)**						
	Middle-grade	0.108	1.895	0.169	1.114	0.955	1.300
	Bottom-grade	0.458	22.019	<0.001	1.580	1.305	1.913
	**Satisfaction with online learning effects (vs. Satisfied)**						
	General	0.352	20.606	<0.001	1.422	1.221	1.655
	Dissatisfied	0.675	42.359	<0.001	1.963	1.602	2.405
	**Online learning time (vs**. **≥** **4 h)**						
	0–2 h	0.521	18.950	<0.001	1.684	1.332	2.129
	2–4 h	0.374	19.125	<0.001	1.453	1.229	1.718
**Stress**							
	**Grade (vs. First-year)**						
	Second-year	0.364	9.596	0.002	1.438	1.143	1.810
	Third–year	0.490	15.423	<0.001	1.633	1.279	2.086
	**Relationship between parents (vs. Good)**						
	General	0.242	2.804	0.094	1.273	0.960	1.690
	Poor	1.102	27.401	<0.001	3.010	1.992	4.547
	**Relationship with father (vs. Good)**						
	General	0.321	5.727	0.017	1.378	1.060	1.793
	Poor	0.914	12.624	<0.001	2.495	1.507	4.132
	**The school records in the class in the past six months (vs. Top-grade)**						
	Middle-grade	−0.119	1.219	0.270	0.888	0.719	1.096
	Bottom-grade	0.462	14.345	<0.001	1.587	1.250	2.015
	**Satisfaction with online learning effects (vs. Satisfied)**						
	General	0.340	9.176	0.002	1.405	1.127	1.750
	Dissatisfied	1.118	73.358	<0.001	3.058	2.368	3.950
	**Online learning time (vs**. **≥4 h)**						
	0–2 h	0.617	18.028	<0.001	1.853	1.394	2.463
	2–4 h	0.343	9.109	0.003	1.409	1.128	1.760

The risk of anxiety was significantly increased among medical students with the following variables: males (OR = 1.421, 95% CI: 1.235–1.634), four-year programs (OR = 1.224, 95% CI: 1.068–1.403), the relationship between parents (general: OR = 1.395, 95% CI: 1.124–1.732), relationship with father (poor: OR = 3.609, 95% CI: 2.252–5.784; general: OR = 1.289, 95% CI: 1.054–1.576), the school records in the past six months (bottom: OR = 1.580, 95% CI: 1.305–1.913), satisfaction with online learning effects (dissatisfied: OR = 1.963, 95% CI: 1.602–2.405; general: OR = 1.422, 95% CI: 1.221–1.655), and online learning time (0–2 h: OR = 1.684, 95% CI: 1.332–2.129; 2–4 h: OR = 1.453, 95% CI: 1.229–1.718) (Table 4).

The risk of stress was significantly increased among medical students with the following variables: grade (second-year: OR = 1.438, 95% CI: 1.143–1.810; third-year: OR = 1.633, 95% CI: 1.279–2.086), the relationship between parents (poor: OR = 3.010, 95% CI: 1.992–4.547), relationship with father (poor: OR = 2.495, 95% CI: 1.507–4.132; general: OR = 1.378, 95% CI: 1.060–1.793), the school records in the past six months (bottom: OR = 1.587, 95% CI: 1.250–2.015), satisfaction with online learning effects (dissatisfied: OR = 3.058, 95% CI: 2.368–3.950; general: OR = 1.405, 95% CI: 1.127–1.750), and online learning time (0–2 h: OR = 1.853, 95% CI: 1.394–2.463; 2–4 h: OR = 1.409, 95% CI: 1.128–1.760) (Table 4).

## Discussion

Public health emergencies not only have a direct impact on individual life and social economy, but also may lead to individual psychological stress reaction, which threatens social stability and economic development (30). COVID-19 is a large-scale worldwide disaster in the 21st century. In a US study, depressive symptoms were found to have increased from 8.5% before COVID-19 to 27.8% during the outbreak (31). Evidence from a systematic review and meta-analysis concluded that COVID-19 pandemic had a negative impact on the mental health of the global population, particularly medical workers and quarantined persons (32). A cross-sectional survey among the Iranian population found that compared with community population, medical students had higher scores of stress, anxiety and depression due to lower experience. At the same time, the compulsory measures from face-to-face teaching to completely online teaching have become new challenges for medical students (33). A comprehensive and large-scale study from 62 countries (30,383 students) in the transition to one learning showed that students were mainly concerned their future professional career and studies, and experienced boredom and frustration (34). In order to formulate the targeted psychological interventions, it is essential to focus on the levels and associated factors of mental health among medical students engaged in online learning during the pandemic.

In the present study, the prevalence of depression, anxiety, and stress symptoms among medical students during online learning were 31.9, 32.9, and 14.6%, respectively, which was significantly higher than the prevalence of students during the non-epidemic period in China (35, 36). Our finding is different from a previous study which showed the prevalence of depression and anxiety was 43.77 and 20.60%, respectively, during the COVID-19 pandemic (37). In that study, Patient Health Questionnaire-9 items (PHQ-9) and Self-Rating Anxiety Scale (SAS) which is different from our study were used to measure the mental health of college students, and only three-fifths (59.52%) were medical majors. Due to the difference in education, the negative impact of the COVID-19 pandemic on medical students and non-medical students is different (38). The different mental health levels among college students in different studies might be related to different psychological scales and different types of students (39). As we all know, the severity of the pandemic varies in different countries, which may cause different degrees of negative psychological impact. An online questionnaire survey conducted in April 2020 among university students in Malaysia utilizing the DASS 21 reported that the prevalence of depression, anxiety, and stress were 29.4, 51.3, and 56.5%, respectively, which were significantly different from our study (40). Combined with the above research results, the COVID-19 pandemic had a negative impact on the mental health of college students, especially medical students. It is important to note that 4.4, 7.2, and 2.7% of medical students reported that the symptoms of depression, anxiety, and stress were at severe and extremely severe levels. While college students with severe depression and anxiety symptoms were at increased risk of suicidal behavior (41). The study reported by Aristovnik et al. concluded that online learning students were more likely to interrupt their studies and felt more socially isolated, compared with students receiving traditional education (34). Social isolation and loneliness increased the risk of depression and anxiety (42). This cross-sectional survey was nearing the end of online learning. Students often face more serious pressure at the end of their study, mainly from the pressure of examination (43). Therefore, it is necessary for colleges and universities to pay special attention to the students whose symptoms were at severe and extremely severe levels and carry out targeted psychological interventions to prevent students from committing suicide.

Multi-factor analysis in our study found that the risk of depression and anxiety was significantly increased among males, which was consistent with the findings from previous studies (28, 44, 45) Online learning at home requires greater self-discipline to complete online courses, particularly in the earlier period when students are adapting to the new system (34). Self-regulation and self-management ability are very important in the process of online learning (46). While, the self-management ability of males was lower than that of females (47). Compared with females, males may be less adaptable to online learning. According to a study, males showed higher psychological pressure during online learning (48). This survey was conducted in June, one month before the final exam for medical students. Several studies reported that most of medical students had exam anxiety (43–49). For the above reasons, males are more likely to experienced negative emotions such as anxiety and depression than females engaged in online learning period in the pandemic. Thus, we need to pay special attention to males. Compared with first-year students, second-year and third-year students experienced higher depression and stress, in agreement with other study (50). It may be related to the fact first-year students mainly study general education courses, and senior students mainly study professional basic and education courses, which is much more difficult than general education courses. The transition from pre-clinical to clinical year is a major turning point, resulting in significant changes in students' learning needs and teaching mode (51). Senior students experienced higher symptoms of depression and stress, which may be due to the improper adjustment of basic courses of online learning, resulting in more worry about the final exam. A study from China reported that compared with first-year students, second-year and third-year students were less satisfied with teachers' teaching effectiveness and teaching methods and arrangements (48). The study also found that the risk of depression and anxiety was significantly increased among medical students with four-year school year. The four-year program has short professional years, many types of medical courses, and great difficulty in course learning. At the same time, these difficulties are exacerbated by the unadaptability brought by the sudden online learning. Finally, it has a negative impact on the mental health of medical students. Online learning environment support was critical for relieving the negative impacts of COVID-19 on the psychosocial health among medical students (50). During the special period of online learning, schools should pay attention to psychological counseling and provide crisis-oriented psychological services, especially the following groups of people: males, students with four-year school year, and senior students.

Family is generally considered to be one of the most influential factors in the social and psychological development of adolescents (52, 53). Based on this research, several family variables (relationship with mother, with father, and between parents) seem to be related to the depression, anxiety, and stress symptoms among medical students engaged in online learning period in the pandemic. The parent-child relationship plays a relatively key role in the development of the individual's psychological well-being and the cultivation of a good personality (54). A systematic review from 100 eligible studies indicated that social support was associated with depression protection, and parental support was the most important among adolescents (20). A harmonious parent-child relationship was more conducive to the cultivation of students' positive academic emotions, which was beneficial to their mental health (55). During this special period of online learning in the pandemic, parents, especially fathers, should pay more attention to students' thoughts and be aware of their psychological needs. At the same time, parents should communicate more with their children, so as to reduce students' mental health problems in special periods.

Our findings indicated that the students' daily online learning time was associated with the depression, anxiety, and stress. Medical students who study online 0–2 and 2–4 h a day had higher negative emotions. The short online learning time results in less communication between students and teachers and less communication between students and students (6, 19). A recent study conducted in Switzerland on 236 students indicated that less social contact has a negative psychological impact on students during the COVID-19 pandemic (56). The present study also demonstrated that as students' satisfaction with online learning effects decreased, the prevalence of symptoms of depression, anxiety, and stress were found to rise both in males and females, suggesting that teachers' online learning effects has a significant impact on students' negative emotions. The transition to online learning occurred suddenly, which is a new challenge for both teachers and students (57). In offline teaching, teachers can communicate face-to-face with students, observe students' listening status at any time, and adjust the speed and content of lectures in time according to students' understanding of the course, which ensure the effective transmission of knowledge (58). Students who used to the traditional face-to-face classroom teaching method can not fully accept online teaching in a short time. In a cross-sectional study of 243 medical students found that 22.3% of medical students perceived severe stress as they did not prefer online learning and had difficulties in time management (51). A cross-sectional survey from more than 13 medical schools in Libya showed that most medical students (64.7%) did not think that online learning could be easily implemented during the COVID-19 pandemic (59). An online survey among 7,084 Chinese college students indicated that percentage of psychological pressure among college students who were unfamiliar with the operation of the learning platform (32.9%) were higher than that of students who were proficient (48). Another cross-sectional survey of 99,559 valid samples from 90 medical schools in China revealed that students' satisfaction with their current online education was positively correlated with their previous learning experiences (60). In China, the vast majority of medical students had online learning experience, and 36.84 and 35.00% of students were most familiar with recorded broadcast courses and MOOCs (60). Therefore, teachers should choose the teaching platform familiar to students for online teaching, so as to improve the quality of online medical education. In Chinese medical education, teachers prefer traditional teaching methods, such as face-to-face teaching (60). Online education has attracted global attention, but it is not common in China (61). Therefore, in order to continuously and effectively carry out online medical education and improve students' satisfaction with online learning, schools should provide more support and training in teachers' teaching. Before carrying out online teaching, teachers should master the various functions of online teaching platform. At the same time, for students, schools should provide more training to improve their self-management ability.

## Limitations

There are limitations that should be considered when interpreting these findings. First, this study was cross-sectional, unlike a longitudinal design, which cannot explain the causal relationships among the study variables. Second, students completed the questionnaire online, which reduced the the return rate of the questionnaire. In addition, information bias is inevitable, although we explained the purpose and significance of this survey to students before the survey. Third, all information was obtained from a self-reported questionnaire, which may lead to recalling bias and reporting bias. Fourth, during the pandemic period, The Ministry of Education of the People's Republic of China launched a policy called “Disrupted classes, undisrupted learning” to provide online learning in Colleges and universities across the country for the first time. In the whole process of using online teaching in Colleges and universities, there may be differences in school level, students' autonomous learning ability, and implementation scheme. The results of this study come from one medical college, and the extrapolation of the results is not necessarily applicable to non-medical students. A nationwide survey should be conducted to compare several provinces, including urban and rural areas in the future. Meanwhile, the COVID-19 pandemic is still ongoing and online learning is still an important means. Prospective studies should be used to explore the influencing factors of college students' mental health in the future.

## Conclusion

In conclusion, during the online learning period in the pandemic, nearly one-third of medical students survived with varying degrees of depression, anxiety, and stress symptoms. Some individual characteristics (gender, grade, length of schooling), family environment (relationship with father, relationship between parents), and online learning environment (students' daily online learning time, and students' satisfaction with online learning effects) play vital roles in medical students' mental health. This study implies that families and schools should pay attention to the negative emotions among medical students engaged in online learning at home during the pandemic, and provide targeted psychological counseling to high-risk students (male, second-year and third-year, four-year program) to alleviate the negative emotions during the pandemic. The findings of this study can provide reference for educators to cope with the psychological problems and formulate the mental health curriculum construction among medical students during online learning.

## Data Availability Statement

The raw data supporting the conclusions of this article will be made available by the authors, without undue reservation.

## Ethics Statement

The studies involving human participants were reviewed and approved by School of Public Health of Wannan Medical College (LL-2020BH2086). Written informed consent to participate in this study was provided by the participants' legal guardian/next of kin. Written informed consent was obtained from the minor(s)' legal guardian/next of kin for the publication of any potentially identifiable images or data included in this article.

## Author Contributions

W-wC: conceptualization, methodology, investigation, writing-original draft, and supervision. L-xS: methodology, investigation, and writing-original draft. LZ: methodology, investigation, and writing-original draft. J-gY: project administration. Y-lJ: methodology, investigation, resources, and project administration. All authors contributed to the article and approved the submitted version.

## Funding

This study was supported by Talent Project of Education Department of Anhui Province (gxfx2017070; gxyqZD2017066), Anhui Province Quality Engineering (2020jyxm2086; 2018JYXM1275; 2015zjjh017), Anhui Province philosophy and social science planning general project (AHSKY2018D55), and Talents Program for Academic Leaders and Reserve Candidates of Wannan Medical College (No. School Administration Letter [2021] No. 46).

## Conflict of Interest

The authors declare that the research was conducted in the absence of any commercial or financial relationships that could be construed as a potential conflict of interest.

## Publisher's Note

All claims expressed in this article are solely those of the authors and do not necessarily represent those of their affiliated organizations, or those of the publisher, the editors and the reviewers. Any product that may be evaluated in this article, or claim that may be made by its manufacturer, is not guaranteed or endorsed by the publisher.

## References

[1] Bao Y Sun Y Meng S Shi J Lu L. 2019-nCoV epidemic: address mental health care to empower society. Lancet. (2020) 395:e37–38. 10.1016/S0140-6736(20)30309-3 32043982 PMC7133594

[2] Johns Hopkins University & Medicine. Coronavirus COVID-19 Global Cases by the Center for Systems Science and Engineering at Johns Hopkins. (2020). Available online at: https://coronavirus.jhu.edu/map.html (accessed April 5, 2020).

[3] WHO. WHO Director-General's Opening Remarks at the Media Briefifing on COVID-19 - 11 March 2020. World Health Organization. (2020). Available online at: https://www.who.int/dg/speeches/detail/who-director-general-s-opening-remarks-at-the-media-briefifing-on-covid-19-\protect\T1\textdollar-\protect\T1\textdollar11-march-2020 (accessed March 15, 2020).

[4] National Health Commission of the People's Republic of China. Update on Novel Coronavirus Pneumonia at 24:00 as at 31st May, 2020. (2020). Available online at: http://www.nhc.gov.cn/xcs/yqtb/202005/230cf04dc30149709ddf4114f81688bf.shtml (accessed July 3, 2021).

[5] Gasmi A Noor S Tippairote T Dadar M Menzel A Bjørklund G. Individual risk management strategy and potential therapeutic options for the COVID- 19 pandemic. Clin Immunol Jun. (2020) 215:108409. 10.1016/j.clim.2020.108409 32276137 PMC7139252

[6] Deng SQ Peng HJ. Characteristics of and public health responses to the coronavirus disease 2019 outbreak in China. J Clin Med. (2020) 9:575. 10.3390/jcm9020575 32093211 PMC7074453

[7] China AP,. Department of Education. Important Notice (Notice of education and school teaching line). (2020). Available online at: http://jyt.ah.gov.cn/tsdw/ahsjyxczx/tzgg/39850684.html (accessed March 5, 2020).

[8] Khalil R Mansour AE Fadda WA Almisnid K Aldamegh M Al-Nafeesah A. The sudden transition to synchronized online learning during the COVID-19 pandemic in Saudi Arabia: a qualitative study exploring medical students' perspectives. BMC Med Educ. (2020) 20:285. 10.1186/s12909-020-02208-z 32859188 PMC7453686

[9] Price M Legrand AC Brier ZMF Gratton J Skalka C. The short-term dynamics of posttraumatic stress disorder symptoms during the acute posttrauma period. Depress Anxiety. (2020) 37:313–20. 10.1002/da.22976 31730736 PMC8340953

[10] Kang L Li Y Hu S Chen M Yang C Yang BX. The mental health of medical workers in Wuhan, China dealing with the 2019 novel coronavirus. Lancet Psychiatry. (2020) 7:e14. 10.1016/S2215-0366(20)30047-X 32035030 PMC7129673

[11] Liem A Wang C Wariyanti Y Latkin CA Hall BJ. The neglected health of international migrant workers in the COVID-19 epidemic. Lancet Psychiatry. (2020) 7:e20. 10.1016/S2215-0366(20)30076-6 32085842 PMC7129812

[12] Yang Y Li W Zhang Q Zhang L Cheung T Xiang YT. Mental health services for older adults in China during the COVID-19 outbreak. Lancet Psychiatry. (2020) 7:e19. 10.1016/S2215-0366(20)30079-1 32085843 PMC7128970

[13] Yuan LL Lu L Wang XH Guo XX Ren H Gao YQ. Prevalence and predictors of anxiety and depressive symptoms among international medical students in China during COVID-19 pandemic. Front Psychiatry. (2021) 12:761964. 10.3389/fpsyt.2021.761964 34803770 PMC8599347

[14] Sun Y. Analysis and Follow-Up Study on Related Factors of PTSD in SARS Patients. Taiyuan: Shanxi Medical University (2005).

[15] Deng J Zhou F Hou W Silver Z Wong CY Chang O. The prevalence of depression, anxiety, and sleep disturbances in COVID-19 patients: a meta-analysis. Ann N Y Acad Sci. (2021) 1486:90–111. 10.1111/nyas.14506 33009668 PMC7675607

[16] Kötter T Wagner J Brüheim L Voltmer E. Perceived medical school stress of undergraduate medical students predicts academic performance: an observational study. BMC Med Educ. (2017) 17:256. 10.1186/s12909-017-1091-0 29246231 PMC5732510

[17] Quek TT Tam WW Tran BX Zhang M Zhang Z Ho CS. The global prevalence of anxiety among medical students: a meta-analysis. Int J Environ Res Public Health. (2019) 16:2735. 10.3390/ijerph16152735 31370266 PMC6696211

[18] Ye W Ye X Liu Y Liu Q Vafaei S Gao Y. Effect of the novel coronavirus pneumonia pandemic on medical students' psychological stress and its influencing factors. Front Psychol. (2020) 11:548506. 10.3389/fpsyg.2020.548506 33178063 PMC7591817

[19] Zhou J Yu H. Contribution of social support to home-quarantined Chinese college students' well-being during the COVID-19 pandemic: the mediating role of online learning self-efficacy and moderating role of anxiety. Soc Psychol Educ. (2021) 25:1–20. 10.1007/s11218-021-09665-4 34720666 PMC8543427

[20] Gariépy G Honkaniemi H Quesnel-Vallée A. Social support and protection from depression: systematic review of current findings in Western countries. Br J Psychiatry. (2016) 209:284–93. 10.1192/bjp.bp.115.169094 27445355

[21] Li Y Peng J Tao Y. Relationship between social support, coping strategy against COVID-19, and anxiety among home-quarantined Chinese university students: a path analysis modeling approach. Curr Psychol. (2021) 4:1–16. 10.1007/s12144-021-02334-x 34629830 PMC8487756

[22] Tang W Hu T Hu B Jin C Wang G Xie C. Prevalence and correlates of PTSD and depressive symptoms one month after the outbreak of the COVID-19 epidemic in a sample of home-quarantined Chinese university students. J Affect Disord. (2020) 274:1–7. 10.1016/j.jad.2020.05.009 32405111 PMC7217769

[23] Bolatov AK Seisembekov TZ Askarova AZ Baikanova RK Smailova DS Fabbro E. Online-learning due to COVID-19 improved mental health among medical students. Med Sci Educ. (2020) 31:1–10. 10.1007/s40670-020-01165-y 33230424 PMC7673686

[24] Zhang JY Shu T Xiang M Feng ZC. Learning burnout: evaluating the role of social support in medical students. Front Psychol. (2021) 12:625506. 10.3389/fpsyg.2021.625506 33692725 PMC7937700

[25] Gong Y Xie XY Xu R Luo YJ. Psychometric properties of the Chinese versions of DASS-21 in Chinese college students. Chin J Clin Psychol. (2010) 18:443–6. 10.16128/j.cnki.1005-3611.2010.04.020

[26] Lovibond SH Lovibond PF. Manual for the depression anxiety stress scales. Sydney, New South Wales: Psychology Foundation. (1995). 10.1037/t01004-000

[27] Wang K Shi HS Geng FL Zou LQ Tan SP Wang Y. Cross-cultural validation of the depression anxiety stress scale-21 in China. Psychol Assess. (2016) 28:e88–100. 10.1037/pas0000207 26619091

[28] Alam MD Lu J Ni L Hu S Xu Y. Psychological outcomes and associated factors among the international students living in china during the COVID-19 pandemic. Front Psychiatry. (2021) 12:707342. 10.3389/fpsyt.2021.707342 34483997 PMC8414650

[29] Gu S He Z Sun L Jiang Y Xu M Feng G. Effects of coronavirus-19 induced loneliness on mental health: sleep quality and intolerance for uncertainty as mediators. Front Psychiatry. (2021) 12:738003. 10.3389/fpsyt.2021.738003 34621198 PMC8490824

[30] Barello S Graffigna G. Caring for health professionals in the COVID-19 pandemic emergency: toward an epidemic of empathy in healthcare. Front Psychol. (2020) 11:1431. 10.3389/fpsyg.2020.01431 32581986 PMC7296111

[31] Ettman CK Abdalla SM Cohen GH Sampson L Vivier PM Galea S. Prevalence of depression symptoms in US adults before and during the COVID-19 pandemic. JAMA Netw Open. (2020) 3:e2019686. 10.1001/jamanetworkopen.2020.19686 32876685 PMC7489837

[32] Wu T Jia X Shi H Niu J Yin X Xie J. Prevalence of mental health problems during the COVID-19 pandemic: a systematic review and meta-analysis. J Affect Disord. (2021) 281:91–8. 10.1016/j.jad.2020.11.117 33310451 PMC7710473

[33] Vahedian-Azimi A Moayed MS Rahimibashar F Shojaei S Ashtari S Pourhoseingholi MA. Comparison of the severity of psychological distress among four groups of an Iranian population regarding COVID-19 pandemic. BMC Psychiatry. (2020) 20:402. 10.1186/s12888-020-02804-9 32770975 PMC7414274

[34] Aristovnik A KerŽič D Ravšelj D TomaŽevič N Umek L. Impacts of the COVID-19 pandemic on life of higher education students: a global perspective. Sustainability. (2020) 12:8438. 10.3390/su12208438 34869802 PMC8634691

[35] Cheung DK Tam DKY Tsang MH Zhang DLW Lit DSW. Depression, anxiety and stress in different subgroups of first-year university students from 4-year cohort data. J Affect Disord. (2020) 274:305–14. 10.1016/j.jad.2020.05.041 32469820

[36] Zou P Sun L Yang W Zeng Y Chen Q Yang H. Associations between negative life events and anxiety, depressive, and stress symptoms: a cross-sectional study among Chinese male senior college students. Psychiatry Res. (2018) 270:26–33. 10.1016/j.psychres.2018.09.019 30243129

[37] Zhan H Zheng C Zhang X Yang M Zhang L Jia X. Chinese college students' stress and anxiety levels under COVID-19. Front Psychiatry. (2021) 12:615390. 10.3389/fpsyt.2021.615390 34177635 PMC8222572

[38] Xie L Luo H Li M Ge W Xing B Miao Q. The immediate psychological effects of coronavirus disease 2019 on medical and non-medical students in China. Int J Public Health. (2020) 65:1445–53. 10.1007/s00038-020-01475-3 32910208 PMC7482373

[39] Luo W Zhong BL Chiu HF. Prevalence of depressive symptoms among Chinese university students amid the COVID-19 pandemic: a systematic review and meta-analysis. Epidemiol Psychiatr Sci. (2021) 30:e31. 10.1017/S2045796021000202 33766163 PMC8047400

[40] Moy FM Ng YH. Perception towards E-learning and COVID-19 on the mental health status of university students in Malaysia. Sci Prog. (2021) 104:368504211029812. 10.1177/00368504211029812 34260295 PMC10358609

[41] Guo L Wang W Wang T Li W Gong M Zhang S. Association of emotional and behavioral problems with single and multiple suicide attempts among Chinese adolescents: modulated by academic performance. J Affect Disord. (2019) 258:25–32. 10.1016/j.jad.2019.07.085 31382101

[42] Loades ME Chatburn E Higson-Sweeney N Reynolds S Shafran R Brigden A. Rapid systematic review: the impact of social isolation and loneliness on the mental health of children and adolescents in the context of COVID-19. J Am Acad Child Adolesc Psychiatry. (2020) 59:1218–39.e3. 10.1016/j.jaac.2020.05.009 32504808 PMC7267797

[43] Wang LC Li X Chung KKH. Relationships between test anxiety and metacognition in Chinese young adults with and without specific learning disabilities. Ann Dyslexia. (2021) 71:103–26. 10.1007/s11881-021-00218-0 33615418

[44] Wang C Zhao H. The impact of COVID-19 on anxiety in Chinese university students. Front Psychol. (2020) 11:1168. 10.3389/fpsyg.2020.01168 32574244 PMC7259378

[45] Khan AH Sultana MS Hossain S Hasan MT Ahmed HU Sikder MT. The impact of COVID-19 pandemic on mental health & wellbeing among home-quarantined Bangladeshi students: a cross-sectional pilot study. J Affect Disord. (2020) 277:121–8. 10.1016/j.jad.2020.07.135 32818775 PMC7410816

[46] Alt D Naamati-Schneider L. Health management students' self-regulation and digital concept mapping in online learning environments. BMC Med Educ. (2021) 21:110. 10.1186/s12909-021-02542-w 33596899 PMC7891141

[47] Xu ZY Li MJ. Influencing factors of undergraduate nursing students' autonomous learning ability. J Nurs Sci. (2019) 34:12–5. 10.1186/s12913-016-1423-5 27409075 PMC4943498

[48] Yu L Huang L Tang HR Li N Rao TT Hu D. Analysis of factors influencing the network teaching effect of college students in a medical school during the COVID-19 epidemic. BMC Med Educ. (2021) 21:397. 10.1186/s12909-021-02825-2 34301244 PMC8300986

[49] Khoshhal KI Khairy GA Guraya SY Guraya SS. Exam anxiety in the undergraduate medical students of Taibah University. Med Teach. (2017) 39:S22–6. 10.1080/0142159X.2016.1254749 28103727

[50] Wang J Liu W Zhang Y Xie S Yang B. Perceived stress among Chinese medical students engaging in online learning in light of COVID-19. Psychol Res Behav Manag. (2021) 14:549–62. 10.2147/PRBM.S308497 34017205 PMC8131094

[51] Abdulghani HM Sattar K Ahmad T Akram A. Association of COVID-19 pandemic with undergraduate medical students' perceived stress and coping. Psychol Res Behav Manag. (2020) 13:871–81. 10.2147/PRBM.S276938 33154682 PMC7608141

[52] Bryant RA Edwards B Creamer M O'Donnell M Forbes D Felmingham KL. The effect of post-traumatic stress disorder on refugees' parenting and their children's mental health: a cohort study. Lancet Public Health. (2018) 3:e249–58. 10.1016/S2468-2667(18)30051-3 29731158

[53] Tang S Xiang M Cheung T Xiang YT. Mental health and its correlates among children and adolescents during COVID-19 school closure: the importance of parent-child discussion. J Affect Disord. (2021) 279:353–60. 10.1016/j.jad.2020.10.016 33099049 PMC7550131

[54] Hong W Liu RD Ding Y Oei TP Zhen R. Parents' phubbing and problematic mobile phone use: the roles of the parent-child relationship and children's self-esteem. Cyberpsychol Behav Soc Netw. (2019) 22:779–86. 10.1089/cyber.2019.0179 31747305

[55] Leadbitter K Macdonald W Taylor C Buckle KL; the PACT Consortium*. Parent perceptions of participation in a parent-mediated communication-focussed intervention with their young child with autism spectrum disorder. Autism. (2020) 24:2129–41. 10.1177/1362361320936394 32667223 PMC7539598

[56] Elmer T Mepham K Stadtfeld C. Students under lockdown: comparisons of students' social networks and mental health before and during the COVID-19 crisis in Switzerland. PLoS ONE. (2020) 15:e0236337. 10.1371/journal.pone.0236337 32702065 PMC7377438

[57] Beniczky S Blümcke I Rampp S Shisler P Biesel E Wiebe S. e-learning comes of age: web-based education provided by the international league against epilepsy. Epileptic Disord. (2020) 22:237–44. 10.1684/epd.2020.1157 32597765

[58] Yusoff MSB Hadie SNH Mohamad I Draman N Muhd Al-Aarifin I Wan Abdul Rahman WF. Sustainable medical teaching and learning during the COVID-19 pandemic: surviving the new normal. Malays J Med Sci. (2020) 27:137–42. 10.21315/mjms2020.27.3.14 32684814 PMC7337950

[59] Alsoufi A Alsuyihili A Msherghi A Elhadi A Atiyah H Ashini A. Impact of the COVID-19 pandemic on medical education: medical students' knowledge, attitudes, and practices regarding electronic learning. PLoS ONE. (2020) 15:e0242905. 10.1371/journal.pone.0242905 33237962 PMC7688124

[60] Wang C Xie A Wang W Wu H. Association between medical students' prior experiences and perceptions of formal online education developed in response to COVID-19: a cross-sectional study in China. BMJ Open. (2020) 10:e041886. 10.1136/bmjopen-2020-041886 33122327 PMC7597486

[61] Mayadas AF Bourne J Bacsich P. Online education today. Science. (2009) 323:85–9. 10.1126/science.1168874 19119225

